# Merging images with different central frequencies reduces banding artifacts in balanced steady‐state free precession magnetic resonance cisternography

**DOI:** 10.1002/acm2.12468

**Published:** 2018-10-05

**Authors:** Koji Matsumoto, Hajime Yokota, Hiroki Mukai, Yoshitada Masuda, Takashi Uno, Tosiaki Miyati

**Affiliations:** ^1^ Department of Radiology Chiba University Hospital Chiba Japan; ^2^ Division of Health Sciences Graduate School of Medical Sciences Kanazawa University Kanazawa Ishikawa Japan; ^3^ Diagnostic Radiology and Radiation Oncology Graduate School of Medicine Chiba University Chiba Japan

**Keywords:** balanced steady‐state free precession, banding artifacts, merged images, off resonance

## Abstract

**Purpose:**

The aim of this study was to evaluate the utility of merged balanced steady‐state free precession (bSSFP) magnetic resonance cisternography images.

**Materials and Methods:**

Twenty ears of 10 healthy volunteers (six men, four women; mean age ± standard deviation, 26.7 ± 1.6 yr) and 10 patients (two men, eight women; mean age, 46.3 ± 10.9 yr) with neoplasm around the sella turcica were included. Two different devices (A and B) were used to confirm the versatility of our method for MR devices with different local magnetic field homogeneity. Images with different central frequencies (±10, ±20, ±30, ±40, and ±50 Hz) were merged with the maximum magnitude of corresponding pixels from the images acquired using both devices. Two neuroradiologists visually graded the image quality of 11 sites in the inner ear and three sites around the sella turcica (scale: 0–2) and compared the quality with that of the corresponding basic image (0 Hz).

**Results:**

The image quality was better in merged images of the vestibule, superior semicircular canal (SCC), posterior SCC, and horizontal SCC (*P *= 0.005 to 0.020 mainly at ±40 and ±50 Hz on devices A and B), as well as in merged images of the sella turcica and right cavernous sinus (±50 Hz, *P* = 0.003 and 0.020 on device B, respectively), than it was in the corresponding basic images.

**Conclusions:**

The maximum magnitude merging of images with different central frequencies makes it possible to reduce banding artifacts on bSSFP images without the need for special pulse sequences and image processing programs.

## 1 INTRODUCTION

Magnetic resonance (MR) cisternography using the three‐dimensional (3D) balanced steady‐state free precession technique^1^ (bSSFP) allows the structures of the inner ear, cavernous sinuses, cisternal spaces, and ventricular system to be clearly visualized.^2^, ^3^, ^4^ Alternatively, a 3D fast spin‐echo technique can also be used to visualize the inner ear.^5^, ^6^ Compared to the fast spin‐echo method, the bSSFP technique can achieve a higher signal‐to‐noise ratio and higher resolution within a shorter acquisition time;^7^ however, when using bSSFP, inhomogeneity of the local magnetic field owing to the aerated sinus results in banding artifacts. The bSSFP technique can also reflect gadolinium enhancement. Although this characteristic is useful for visualizing the nerves in the cavernous sinus and tumors near the cistern,^8^ banding artifacts owing to the paranasal sinuses can impede visualization of the cavernous sinus and sella turcica. Hence, methods that can reduce banding artifacts are required to improve the visualization of these structures.

Several methods for reducing banding artifacts have been proposed, for instance, by merging multiple bSSFP acquisitions, each with a different radio frequency (RF) phase increment from excitation to excitation.^9^, ^10^, ^11^, ^12^, ^13^, ^14^, ^15^ However, these methods cannot be used on all MR devices.

In bSSFP MR imaging, the locations of banding artifacts are defined according to the morphology of the imaging target and its surrounding structures. To reduce the banding artifacts in regions with low local magnetic field homogeneity, such as the skull base, we proposed a method of merging bSSFP MR cisternography images with different central frequencies. This idea has been discussed previously in the bSSFP community,^16^ with the aim of obtaining stable functional MR imaging of the brain by extending its spatial coverage in areas with high local magnetic field homogeneity. Changing the central frequency is very similar to changing the phase cycling angle for bSSFP, except that it may excite the wrong position. Moreover, unlike other suggested methods for reducing banding artifacts, our proposed method is widely available, even on old MR machines.

In the present study, we examined the utility and versatility of the merged images in healthy individuals and patients with neoplasm around the sella turcica who underwent gadolinium‐enhanced imaging.

## 2 MATERIALS AND METHODS

This study was approved by the ethics committee at our institution, and written informed consent was obtained from each subject prior to his/her participation.

### 2.A Scanning parameters for the healthy individuals

Twenty ears from 10 healthy adults (six men, four women; mean ± standard deviation age, 26.7 ± 1.7 yr) were evaluated. All MR imaging studies were performed on two 1.5‐T MR devices (device A: Signa HDxt version 15, GE Healthcare, Milwaukee, WI, USA, developed in 2008; device B: Intera Achieva Nova‐Dual release 3.2, Philips Medical Systems, Best, Netherlands, developed in 2010) with 8‐channel head coils. Two different devices were used in order to confirm the versatility of our method for MR machines with different local magnetic field homogeneity. Each subject was scanned using both devices. On device A, images of the inner ear were acquired in the axial plane using the following parameters: pulse sequence, 3D bSSFP (3D‐fast imaging employing steady‐state acquisition); repetition time (TR), 5.6 ms; echo time (TE), 2.4 ms; flip angle, 60°; field‐of‐view (FOV), 180 ×  180 × 112 mm; resolution, 0.7 × 0.63 × 0.8 mm (reconstruction, 0.35 × 0.35 × 0.4 mm); and scan time, 1 min 55 s. On device B, images were acquired in the axial plane for the inner ear and in the coronal plane for the sella turcica using the following parameters: pulse sequence, 3D bSSFP (3D‐balanced fast field echo); TR, 5.0 ms; TE, 2.0 ms; flip angle, 60°; FOV, 180 × 180 × 112 mm; resolution, 0.63 × 0.58 × 0.8 mm (reconstruction, 0.35 × 0.35 ×  0.4 mm); and scan time, 1 min 50 s.

### 2.B Central frequency shift and image merging

**Figure 1:**
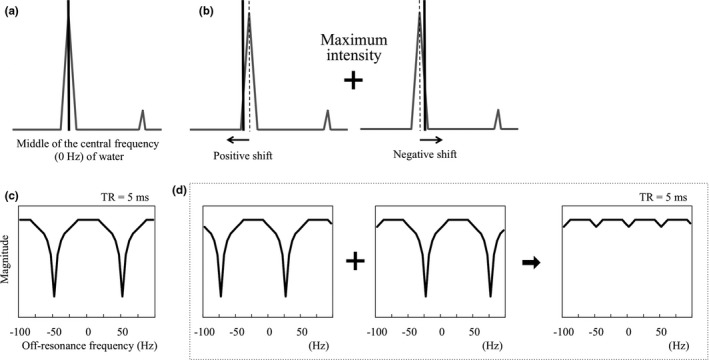
Schematic diagram of the central frequencies of the basic image (a, c) and merged images (b, d). These figures imply that banding appears every 1/(2*TR), i.e., at 100 Hz based on a repetition time (TR) of 5.0 ms. When the center frequency is shifted, the positions of the banding artifacts change. Merged images were obtained using the maximum intensities of the corresponding pixels across each of the different central frequencies.

The volume shimming was set at the cerebellopontine angle to obtain the optimum resonant frequency of water in all healthy volunteers. Basic images were obtained using bSSFP with a central frequency of 0 Hz [Figs. 1(a), 1(c)]. Next, images offset to −50, −40, −30, −20, −10, +10, +20, +30, +40, and +50 Hz with reference to the center frequency of the basic image were obtained. For device B, frequency offset was performed after volume shimming at the same set position due to its characteristics. We selected this range of center frequencies because banding appears every 1/(2 * TR), i.e., at 100 Hz based on a TR of 5.0 ms. Thereafter, images with positive and negative frequencies were merged, respectively [±10, ±20, ±30, ±40, and ±50 Hz; Figs. 1(b), 1(d)]. At that time, each voxel value was computed using the maximum intensity of the corresponding pixels across each of the different central frequencies on an Advantage Workstation Volume Share 5 (GE Healthcare).

### 2.C Visual grading

Two board‐certified neuroradiologists with 11 and 9 yr of experience independently evaluated the anatomical structures, including all four nerves in the internal auditory canal (facial, cochlear, superior vestibular, and inferior vestibular nerves), the cochlear turns, modiolus, spiral lamina of the cochlea, vestibule, and superior, posterior, and horizontal semicircular canals (SCCs) on the basic and merged images.^6^ Each neuroradiologist visually graded the image quality of the anatomical structures as good (grade 2, no artifacts and the entire structure was visible), fair (grade 1, image contained artifacts, but the entire structure was visible), or poor (grade 0, visibility of the structures was impeded by artifacts). Any discrepancies in grading between the two readers were resolved by consensus.

### 2.D Phantom experiment for image quality

**Figure 2:**
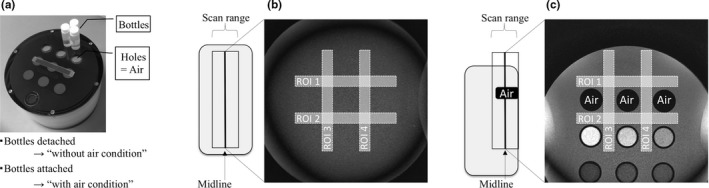
Set up for the phantom experiment. (a) Photograph of the phantom. (b, c) Phantom images with different local magnetic field homogeneity (b, with air; c, without air). The white rectangles depict the regions of interest and their placement for each condition (b, with air; c, without air).

A phantom experiment was performed to investigate the RF excitation efficiency on conditions with and without local magnetic field inhomogeneity. As shown in Fig. 2, we used a cylindrical‐shaped phantom (90‐401 type; NIKKO FINES INDUSRIES Co., Ltd., Tokyo, Japan), which was designed such that three sample bottles could be attached or detached [Fig. 2(a)]. The conditions with and without local magnetic field inhomogeneity were simulated by imaging the phantom with the sample bottles detached [“with air,” Fig. 2(b)] or attached [“without air,” Fig. 2(c)], respectively. Using device B, axial images of the phantom were acquired using the same imaging parameters as those used above for evaluating the internal ear. First, basic images were obtained using bSSFP with a central frequency of 0 Hz. Following this, images offset to −100, −90, −80, −70, −60, −50, −40, −30, −20, −10, +10, +20, +30, +40, +50, +60, +70, +80, +90, and +100 Hz with reference to the center frequency of the basic images were obtained.

We measured the signal‐to‐noise ratio (SNR) of each image. Four rectangular regions of interest (ROI) were drawn on the phantom images. For the without air condition, the four ROIs were placed around the holes [Fig. 2(b)]. The ROIs for the condition without air were the same shape as those in the condition with air but different locations [Fig. 2(c)]. The size of each ROI was 120 × 9 mm. The SNRs of ROI 1, 2, 3, and 4 were calculated as follows:

$$SNR=Mean_{\mathrm{ROI}}/SD_{\mathrm{ROI}}$$

where *Mean*_*ROI*_ and *SD*_*ROI*_ are the mean and standard deviation of the signal intensity of ROI 1, 2, 3, and 4, respectively. Finally, the SNRs of the four ROIs were averaged for each condition.

### 2.E Clinical study of the sella turcica and cavernous sinus

We additionally enrolled 10 patients (two men, eight women; mean age, 46.3 ± 24.1 yr; six, two, and two cases of pituitary adenoma, Rathke's cleft cyst, and tuberculum sellae meningioma, respectively) requiring preoperative gadolinium‐enhanced imaging to evaluate the structures around the sella turcica. Using device B, images of the structures around the sella turcica were acquired in the coronal plane using the same imaging parameters as those used above for evaluating the internal ear. In this clinical study, the basic and −50 Hz images were merged. We selected the basic images because they were necessary for diagnosis. The −50 Hz images were selected based on the results obtained from the healthy individuals. The neuroradiologists evaluated the sella turcica and bilateral cavernous sinuses using the same grading system as that used above for evaluating the internal ear.

### 2.F Statistical analysis

All statistical analyses were performed using R (The R Foundation for Statistical Computing, Vienna, Austria).^17^ The inter‐reader reliability for grading was evaluated using the weighted Cohen's Kappa test. The grade of each merged image with different central frequencies was compared to that of the basic image, respectively, using a Wilcoxon signed‐rank test. *P*‐values were adjusted using the Bonferroni method to correct for multiple comparisons. Statistical significance was set at *P *<* *0.05.

## 3 RESULTS

### 3.A Assessments of the internal ear

**Figure 3:**
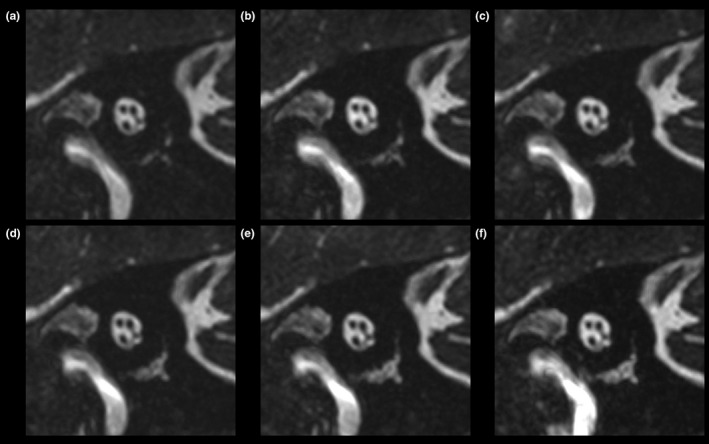
Images of the short axis of the internal auditory canal of a healthy volunteer, acquired using device A. (a) Basic image. (b) ±10 Hz image. (c) ±20 Hz image. (d) ±30 Hz image. (e) ±40 Hz image. (f) ±50 Hz image.

**Figure 4:**
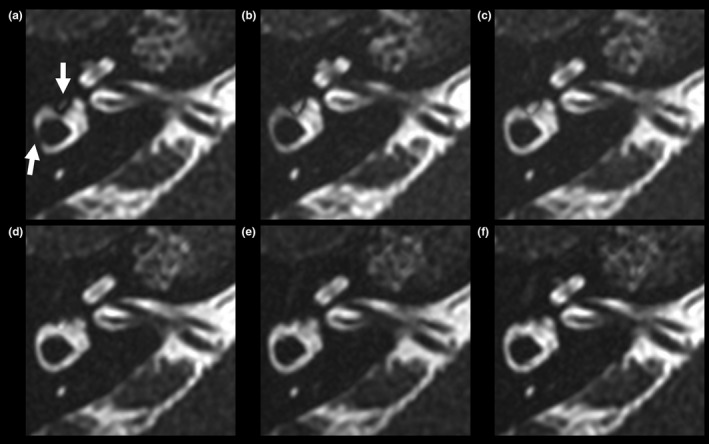
Transverse images of a healthy volunteer, acquired using device A. (a) Basic image. (b) ±10 Hz image. (c) ±20 Hz image. (d) ±30 Hz image. (e) ±40 Hz image. (f) ±50 Hz image. Arrows indicate banding artifacts in the horizontal semicircular canals and vestibule.

**Figure 5:**
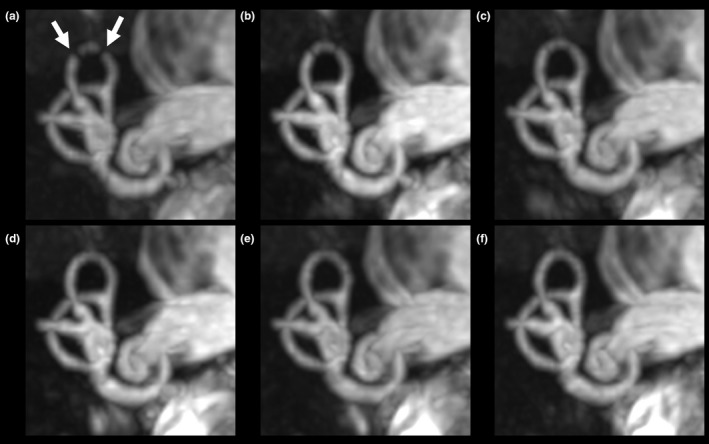
Coronal maximum intensity projection images (18‐mm thick) acquired on device A from a healthy volunteer. (a) Basic image. (b) ±10 Hz image. (c) ±20 Hz image. (d) ±30 Hz image. (e) ±40 Hz image. (f) ±50 Hz image. Arrows indicate banding artifacts in the superior semicircular canals.

Excellent inter‐reader agreement (κ* *= 0.878) was obtained for the grades of all structures. Table 1 shows the results of the comparisons between the basic and merged images. The image quality of all four nerves in the internal auditory canal, modiolus, and spiral lamina was equal to that of the basic images (Figs. 3, 4, 5). In some cases, the merged images demonstrated better quality than did the basic images in the cochlear turns, although this difference was not statistically significant. In the vestibule, the grades of the ±50 Hz images acquired using device B were significantly higher than were those of the basic images (*P *=* *0.005). In the superior SCC, the grades of the ±20 to ±50 Hz images acquired using device A and of the ±50 Hz images acquired using device B were significantly higher than were those of the basic images (*P *=* *0.005 and *P *=* *0.020, respectively) (Fig. 5). In the posterior SCC, the grades of the ±40 Hz images acquired on device B were significantly higher than were those of the basic images (*P *=* *0.020). In the horizontal SCC, the grades of the ±10 to ±50 Hz images acquired using device A and of the ±30 to ±50 Hz images acquired using device B were significantly higher than were those of the basic images (*P *=* *0.020 and *P *=* *0.020, respectively) (Fig. 4).

**Table 1:** Grades of the structures of the inner ear on devices A and B

	Basic	±10 Hz					±20 Hz					±30 Hz					±40 Hz					±50 Hz				
	Mean grade (SD)	Mean grade (SD)	Compared to the basic images				Mean grade (SD)	Compared to the basic images				Mean grade (SD)	Compared to the basic images				Mean grade (SD)	Compared to the basic images				Mean grade (SD)	Compared to the basic images			
			Better	Worse	Equal	*P*		Better	Worse	Equal	*P*		Better	Worse	Equal	*P*		Better	Worse	Equal	*P*		Better	Worse	Equal	*P*
*Device A (n = 20)*																										
Internal auditory canal																										
Facial nerve	1.95 (0.22)	1.95 (0.22)	0	0	20		1.95 (0.22)	0	0	20		1.95 (0.22)	0	0	20		1.95 (0.22)	0	0	20		1.95 (0.22)	0	0	20	
Cochlear nerve	2.00 (0.00)	2.00 (0.00)	0	0	20		2.00 (0.00)	0	0	20		2.00 (0.00)	0	0	20		2.00 (0.00)	0	0	20		2.00 (0.00)	0	0	20	
Superior vestibular nerve	1.95 (0.22)	1.95 (0.22)	0	0	20		1.95 (0.22)	0	0	20		1.95 (0.22)	0	0	20		1.95 (0.22)	0	0	20		1.95 (0.22)	0	0	20	
Inferior vestibular nerve	2.00 (0.00)	2.00 (0.00)	0	0	20		2.00 (0.00)	0	0	20		2.00 (0.00)	0	0	20		2.00 (0.00)	0	0	20		2.00 (0.00)	0	0	20	
Cochlea																										
Cochlear turn	1.65 (0.49)	1.60 (0.60)	1	1	18	1.000	1.75 (0.44)	2	0	18	1.000	1.85 (0.37)	4	0	16	0.625	1.75 (0.44)	4	2	14	1.000	1.70 (0.47)	3	2	15	1.000
Modiolus	2.00 (0.00)	2.00 (0.00)	0	0	20		2.00 (0.00)	0	0	20		2.00 (0.00)	0	0	20		2.00 (0.00)	0	0	20		1.95 (0.22)	0	1	19	1.000
Spiral lamina	0.90 (0.31)	1.10 (0.55)	3	0	17	1.000	1.00 (0.46)	2	0	18	1.000	1.10 (0.55)	3	0	17	1.000	0.90 (0.31)	0	0	20		1.00 (0.46)	2	0	18	1.000
Vestibule	1.05 (0.22)	1.05 (0.22)	0	0	20		1.05 (0.22)	0	0	20		1.05 (0.22)	0	0	20		1.20 (0.41)	3	0	17	1.000	1.25 (0.44)	4	0	16	0.625
Semicircular canals																										
Superior	0.90 (0.72)	1.15 (0.67)	5	0	15	0.313	1.50 (0.51)	11	0	9	0.005^a^	1.55 (0.51)	12	0	8	0.002^a^	1.65 (0.49)	14	0	6	< 0.001^a^	1.65 (0.49)	13	0	7	0.001^a^
Posterior	1.40 (0.88)	1.50 (0.69)	3	1	16	1.000	1.50 (0.51)	6	4	10	1.000	1.70 (0.47)	5	2	13	1.000	1.80 (0.41)	6	0	14	0.156	1.70 (0.47)	5	2	13	1.000
Horizontal	1.10 (0.79)	1.60 (0.68)	9	0	11	0.020^a^	1.80 (0.41)	12	0	8	0.002^a^	1.75 (0.44)	11	0	9	0.005^a^	1.90 (0.31)	13	1	6	0.007^a^	1.75 (0.44)	12	1	7	0.015^a^
*Device B (n = 20)*																										
Internal auditory canal																										
Facial nerve	1.95 (0.22)	1.95 (0.22)	0	0	20		1.90 (0.31)	0	1	19	1.000	1.95 (0.22)	0	0	20		1.95 (0.22)	0	0	20		1.95 (0.22)	0	0	20	
Cochlear nerve	2.00 (0.00)	2.00 (0.00)	0	0	20		1.95 (0.22)	0	1	19	1.000	2.00 (0.00)	0	0	20		2.00 (0.00)	0	0	20		2.00 (0.00)	0	0	20	
Superior vestibular nerve	1.95 (0.22)	1.90 (0.31)	0	1	19	1.000	1.85 (0.37)	0	2	18	1.000	1.90 (0.31)	0	1	19	1.000	1.90 (0.31)	0	1	19	1.000	1.90 (0.31)	0	1	19	1.000
Inferior vestibular nerve	2.00 (0.00)	2.00 (0.00)	0	0	20		1.90 (0.31)	0	2	18	1.000	2.00 (0.00)	0	0	20		1.95 (0.22)	0	1	19	1.000	1.95 (0.22)	0	1	19	1.000
Cochlea																										
Cochlear turn	1.75 (0.44)	1.70 (0.47)	0	1	19	1.000	1.80 (0.41)	1	0	19	1.000	1.90 (0.31)	3	0	17	1.000	1.95 (0.22)	4	0	16	0.625	2.00 (0.00)	5	0	15	0.313
Modiolus	2.00 (0.00)	2.00 (0.00)	0	0	20		2.00 (0.00)	0	0	20		2.00 (0.00)	0	0	20		2.00 (0.00)	0	0	20		2.00 (0.00)	0	0	20	
Spiral lamina	1.60 (0.50)	1.60 (0.50)	0	0	20		1.55 (0.51)	0	1	19	1.000	1.70 (0.47)	2	0	18	1.000	1.70 (0.47)	2	0	18	1.000	1.70 (0.47)	2	0	18	1.000
Vestibule	1.00 (0.00)	1.00 (0.00)	0	0	20		1.05 (0.22)	1	0	19	1.000	1.15 (0.37)	3	0	17	1.000	1.20 (0.41)	4	0	16	0.625	1.55 (0.51)	11	0	9	0.005^a^
Semicircular canals																										
Superior	1.00 (0.79)	0.95 (0.76)	2	3	15	1.000	1.35 (0.49)	8	1	11	0.195	1.60 (0.51)	9	1	10	0.068	1.55 (0.51)	9	2	9	0.161	1.70 (0.47)	9	0	11	0.020^a^
Posterior	1.40 (0.75)	1.45 (0.76)	2	1	17	1.000	1.80 (0.41)	9	1	10	0.107	1.75 (0.44)	7	2	11	0.606	1.95 (0.22)	9	0	11	0.020^a^	1.90 (0.31)	9	2	9	0.195
Horizontal	1.15 (0.88)	1.30 (0.66)	5	2	13	1.000	1.60 (0.50)	10	2	8	0.166	1.75 (0.44)	9	0	11	0.020^a^	2.00 (0.00)	11	0	9	0.005^a^	1.95 (0.22)	11	1	8	0.017^a^

a *P* < 0.05. SD, standard deviation.

**Figure 6:**
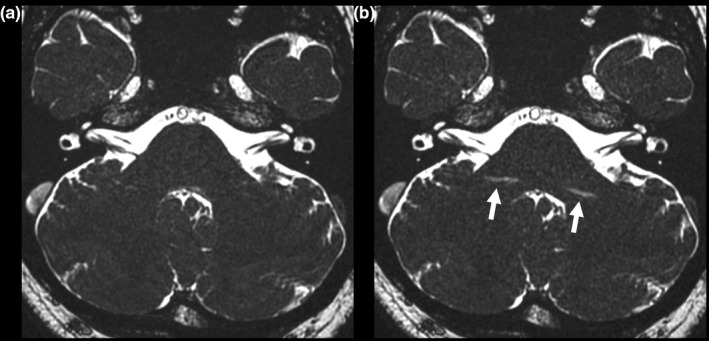
Images of a healthy volunteer acquired using device A, with wrapping artifacts in the slice‐encoding direction (arrows). (a) ±30 Hz image. (b) ±50 Hz image.

Some cases had obvious wrapping artifacts in the slice‐encoding direction on the merged images with large differences in the central frequencies (Fig. 6). For example, in the ±50 Hz image, wrapping artifacts were observed in six and one image(s) from the 10 individuals obtained using devices A and B, respectively.

### 3.B Phantom experiment

**Figure 7:**
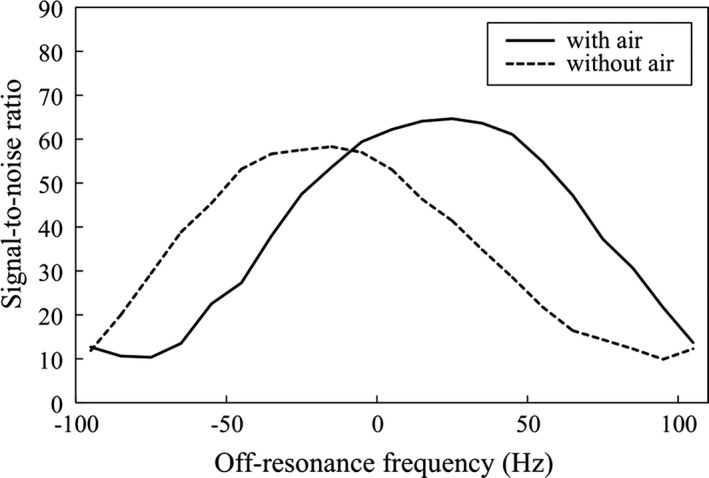
Signal‐to‐noise ratio (SNR) of the original image obtained from the phantom experiment.

As shown in Fig. 7, among the images offset from −100 Hz to +100 Hz, the phantom images offset at 20 Hz and −20 Hz showed the maximum SNR value for the with and without air conditions, respectively. Therefore, the −10 to +40 Hz and −50 to 0 Hz phantom images for the with and without air conditions, respectively, surpassed 90% of the maximum value.

### 3.C Assessments of the sella turcica and cavernous sinus from the clinical study

**Figure 8:**
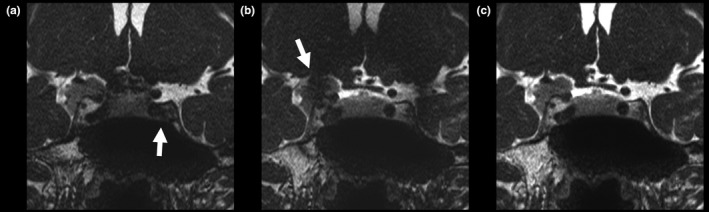
Contrast‐enhanced balanced steady‐state free precession images of a tuberculum sellae meningioma in a 50‐yr‐old woman. (a) Basic image. (b) −50 Hz image. (c) Merged image. Although banding artifacts are visible in the basic and −50 Hz images (arrows), they are less visible on the merged image.

Table 2 summarizes the findings from the sella turcica and cavernous sinus. No grading discrepancies were noted between the two readers when evaluating these structures (κ* *= 1.000). On the basic images, linear, or arcuate artifacts were clearly visualized near the sinuses of all 10 patients (Fig. 8). In 9 of the 10 patients, the sella turcica received a grade of 0 on the basic images because of artifacts from the sphenoid sinus. In the remaining patient, the sphenoid sinus was hypoplastic. In six and four of the 10 patients, the right and left cavernous sinuses, respectively, were classified as grade 0 on the basic images. In contrast, the merged images showed fewer artifacts and the structures were scored as grade 2. The grades of both the sella turcica and right cavernous sinus were significantly higher on the merged images than they were on the basic images (*P *=* *0.003 and *P *=* *0.020, respectively).

**Table 2:** Grades of the structures around the sella turcica on device B

	Basic	Merge of basic and −50 Hz				
	Mean grade (SD)	Mean grade (SD)	Compared to the basic images			
			Better	Worse	Equal	*P*
Device B (*n* = 10)						
Sella turcica	0.20 (0.63)	2.00 (0.00)	9	0	1	0.003^a^
Right cavernous sinus	0.80 (1.03)	2.00 (0.00)	6	0	4	0.020^a^
Left cavernous sinus	1.20 (1.03)	2.00 (0.00)	4	0	6	0.072

a *P* < 0.05. SD, standard deviation.

## 4 DISCUSSION

In the present study, we merged the maximum intensities of each pixel corresponding to different central frequencies to determine whether this method would reduce the banding artifacts on MR cisternography images. Indeed, we found that this approach reduced the banding artifacts and improved visualization of the inner ear on bSSFP MR cisternography images. Furthermore, our method can be applied clinically not only to the inner ear structures but also to other parts of the body, such as the skull base including the sella turcica and cavernous sinus, two structures that are adjacent to aerated tissue, and thus are likely to show banding artifacts. Using the bSSFP technique, the large matrix size required leads to a prolonged TR, which causes banding artifacts in the bSSFP sequence. Our method of merging images reduced these banding artifacts, allowing us to acquire high‐resolution images, even when using older MR devices.

Several methods have been developed to reduce banding artifacts, including maximum‐intensity SSFP (MI‐SSFP),^11^, ^12^ magnitude‐sum SSFP,^13^ complex‐sum SSFP,^14^ and sum‐of‐squares SSFP.^15^ The basic mechanism of these methods is to acquire and merge images that have different banding artifact locations owing to the use of different RF phases, and our approach was based on a similar concept. Among the above methods, MI‐SSFP, such as constructive interference in the steady state, has become relatively popular. Using MI‐SSFP, images with different RF phases are independently acquired and the final images are formed by assigning each pixel the maximum magnitude of the corresponding pixels across the images. However, these methods cannot be used on all MR devices. Therefore, one advantage of our method is its versatility, as the central frequency can be adjusted even on older devices.

Basic images of internal structures, such as the internal auditory canal and modiolus, were not affected by banding artifacts and were of good image quality. Therefore, a technique for removing banding artifacts might not always be necessary for these structures. Shifting the central frequency might adversely affect the selectivity of the excitation slice, causing positional deviation of the FOV. However, no such issue occurred in the present study because the structure of the inner ear was clearly delineated even in ±50 Hz images. By contrast, many banding artifacts occurred in the basic images of the vestibule, superior SCC, and horizontal SCC. The reason is that aerated tissues, including the mastoid sinus, are located near the outer structure of the inner ear. This factor can cause inhomogeneity in the local magnetic field and an increased incidence of banding artifacts.

Although, like the images of the vestibule, the basic images of the superior and horizontal SCCs were affected by banding artifacts, the banding artifacts in the images of the SCCs could be removed using low‐frequency‐shift merged images while the artifacts in the vestibule images could not. The superior and horizontal SCCs are located more outward than the vestibule in the temporal bone, implying that the SCCs are in areas with stronger local magnetic field inhomogeneity than the vestibule. In general, the more uneven the local magnetic field become, the more easily the position of the banding artifacts changed. Additionally, in our phantom experiments, the ranges of suitable frequency offset were different between the conditions with and without local magnetic field inhomogeneity. Our method of using the off‐resonance frequency was sensitive to the local magnetic field inhomogeneity, and thus it might be realized by freely setting the frequency according to the inhomogeneity of the local magnetic field in clinical settings.

Here, we noted that fewer banding artifacts were observed on high‐frequency‐shift merged images than on low‐frequency‐shift merged images. However, the greater the center frequency shift, the lower the excitation efficiency of the FOV. In addition, wrapping artifacts in the slice encoding direction accompany large central frequency shifts. Hence, in order to reduce banding artifacts, this study showed the necessity of using the minimum frequency shift while recognizing these two factors.

Since our method merges multiple images, it can be applied to a region where there is no influence of motion between the images, for instance during spinal cord MR myelography.^18^ Banding artifacts are especially likely to occur when imaging the cervical spinal cord and thoracic cord owing to local magnetic field inhomogeneity, and therefore our method will likely be highly useful in clinical settings. Other applications include imaging of the musculoskeletal regions, particularly articular cartilage^19^ and shoulder joint MR arthrography.^20^

Several limitations of our study should be noted. First, the scan time of this study was extended to 3 min 50 s because both positive and negative images were needed, which is twice as long as the usual scan time of 1 min 55 s. Longer scan times might result in motion artifacts and mismatched images during merging. However, we did not encounter any problems during the image fusion procedures in this study, and sufficient images were obtained even for small structures of the inner ear. Moreover, the longer scan time in our study was similar to those used in other studies, as many SSFP techniques require long scan times to acquire multiple images using different RF phases. Second, no comparison was made to RF‐based methods (constructive interference in the steady state‐type sequences). However, since our study aimed to confirm the versatility of our method, we used two devices with different local magnetic field homogeneities. Device B was an MR device in which RF‐based methods could not be applied. Third, the small sample size of the patient group might limit the generalizability of the results to clinical settings. However, our results showed statistically significant differences, implying that the statistical test had enough power. Finally, the developmental degrees of the aerated sinuses were not evaluated individually, and differences in these areas might have affected our results. However, we selected healthy volunteers and patients with disorders that do not interrupt the aerated sinuses, such as chronic otitis media. These participants should exhibit the artifacts that are typical of aerated sinuses. Therefore, we believe that the results of our study are robust.

## 5 CONCLUSIONS

The maximum magnitude merging of images with different central frequencies makes it possible to reduce banding artifacts on bSSFP MR cisternography images without the need for special pulse sequences and image processing programs.

## CONFLICT OF INTEREST

The authors disclose no conflict of interest.
